# Prevalence and Pattern of Pulmonary Manifestation in Patients with Connective Tissue Disorder

**DOI:** 10.7759/cureus.7618

**Published:** 2020-04-10

**Authors:** Assadullah Dahani, Shafique R Arain, Amir Riaz, Furqan Khan, Rakhshanada Jabeen

**Affiliations:** 1 Rheumatology, Jinnah Postgraduate Medical Centre, Karachi, PAK; 2 Rheumatology, Liaquat National Hospital and Medical College, Karachi, PAK; 3 Internal Medicine, Jinnah Postgraduate Medical Centre, Karachi, PAK

**Keywords:** connective tissue diseases, pulmonary manifestations, interstitial lung disease

## Abstract

Background

A substantial number of deaths and morbidities are caused by pulmonary involvement in connective tissue disorders (CTDs). The majority of patients suffering from CTDs show diverse clinical presentations arising in the lungs, the diaphragm, vasculature, and the pleura during the course of their disease. The clinical course is highly variable ranging from asymptomatic to symptomatic and from reversible to an irreversible stage. The most common occurring pulmonary appearance is the interstitial disease of the lung. In our study, we aim to determine the prevalence and pattern of pulmonary manifestation in patients with CTD presenting at a tertiary care hospital of Karachi, Pakistan.

Methods

A descriptive cross-sectional study was conducted in the department of Rheumatology Medical Unit II, Jinnah Postgraduate Medical Centre (JPMC), Karachi, Pakistan. All patients having CTD were enrolled. Patients with human immunodeficiency virus (HIV) infection, smokers, chronic obstructive pulmonary disease (COPD), bronchogenic carcinoma and patients with underlying cardiac disease were excluded from the study. Pulmonary manifestations like nonspecific interstitial pneumonia (NSIP), pleural effusion, usual interstitial pneumonia (UIP), interstitial pneumonitis, pulmonary arterial hypertension (PAH), tuberculosis, and pulmonary embolism were observed. In addition to this, treatment history and immunological test findings of these patients were also recorded.

Results

A total of 290 patients with rheumatologic disorders were included in this study, out of which 90.7% (263) were females and the average age of the entire cohort was 35.13 ± 12.53 years. A majority, 71% (206), of the patients were young (≤ 40 years of age). Rheumatoid arthritis (RA) was the most commonly observed rheumatologic disorder in this cohort which was in 70.3% (204) patients. RA was more prevalent in patients above 40 years of age as compared to young patients (≤ 40 years of age) with a frequency of 81% vs. 66%; p=0.012. Pulmonary involvement was observed in 27.2% of the patients while no pulmonary involvement was noted in 72.8% of patients.

Conclusion

Pulmonary manifestations in patients with CTD is not that uncommon (27.2%). PAH and NSIP collectively account for three-fourth of the total prevalent cases of pulmonary involvement in CTD patients.

## Introduction

Pulmonary manifestations are prevalent and are significant determinants in morbidity and mortality of connective tissue disorders (CTDs) [1,2]. Lung disease may be pleomorphic in this setting as any lung compartment may be involved [1-3]. During the course of the disease, most patients with CTDs show signs of involvement of the lung, vasculature, pleura, and diaphragm [4].

In patients with scleroderma, interstitial lung disease (ILD) was observed in 72% to 80% of the patients while it was 4%-70% in rheumatoid arthritis (RA) and 5% to 97% in polymyositis [5,6]. In over half of the patients with systemic lupus erythematosus (SLE), respiratory tract involvement takes place during their disease [4]. Pulmonary manifestations in RA are diverse with pleural involvement being the most common. In patients with scleroderma, the most common pulmonary events are interstitial fibrosis and pulmonary hypertension [7,8]. There is evidence suggesting that the occurrence of ILD increases in patients who are suffering from CTDs. Studies carried out recently have shown that radiographic occurrence percentages of subclinical ILD range between 30% and 60%. Diseases of the connective tissue are often linked with diseases of the lung that result in causing high rates of mortality and morbidity including the following: disease of the airway, diseases of the interstitial nature, pleural involvement, and diseases of the vascular nature [1,9].

For the detection of the disease and possible lesions that may be reversed, high-resolution computed tomography (HRCT) is often utilized, thereby, aiding physicians in the overall diagnosis and management of the disease. The involvement of the lungs in CTDs contributes a large percentage in the overall morbidity and mortality of the patients [10].

Histologically pneumonitis is the most common pattern seen among the various types of ILD. Given that the majority of these patients have a compromised immune system, infections are the leading reason behind diseases of the respiratory system [11]. Consequently, negative effects that may be caused by different medications such as methotrexate and leflunomide should also be considered in the overall diagnosis. Because of this reason, the treatment, prognosis, and management of the disease is rather difficult and must, therefore, be based on combining multiple methodologies including symptoms, findings on the laboratory and use of the imaging techniques [12]. For detecting interstitial disease of the lung, X-ray of the chest is utilized, however, it is not so sensitive. On the other hand, HRCT is highly sensitive and specific and is much more useful in providing a diagnosis of interstitial disease. Additionally, HRCT provides images that may be formatted again; they are relatively cheaper and non-invasive [12,13].

Over the previous years, patients suffering from CTDs are now examined for patterns of radiology and findings of the pathological nature are also used in idiopathic interstitial pneumonia. Depending on the extent and severity of the disease, there are various treatment options available which include administration of corticosteroid, immunosuppressant, biologic disease-modifying anti-rheumatic drugs (DMARDs) and other medications. Very few local studies were conducted to see the pulmonary involvement in CTDs and this is the first exclusive and comprehensive study in our population to determine the prevalence and pattern of pulmonary manifestations in patients with CTDs.

## Materials and methods

A descriptive cross-sectional study was performed from August 1, 2018 to April 30, 2019 at the department of Rheumatology Medical Unit II Karachi, Pakistan. Institutional approval was obtained from the department before the commencement of the study. Furthermore, signed informed consent was also obtained from all study participants after explaining the pros and cons of the study. All patients with CTD were consecutively enrolled. The study excluded patients with human immunodeficiency virus (HIV), chronic obstructive pulmonary disease (COPD), bronchogenic carcinoma, smokers, and patients with an underlying cardiac disease based on history and clinical examinations. Clinical history, examination, baseline tests such as X-ray chest and electrocardiography (ECG) were performed. Echocardiography and HIV serology (after taking consent) were done only in those patients whose clinical presentation and baseline tests were suggesting further testing. As a regular practice, the antinuclear antibody (ANA) test profile was obtained for all the patients by the immunoblot method which included 17 important autoantibodies typical of various CTDs, namely ANA, anti-double-stranded DNA antibody (anti-dsDNA), anti-Smith (anti-Sm), anti-ribonucleoprotein antibodies (anti-RNP), anti-Ro, anti-La, antitopoisomerase 1 (anti-Scl-70), anti-Mi-1, antineutrophil cytoplasmic antibody (c-ANCA), perinuclear anti-neutrophil cytoplasmic antibody (p-ANCA), anti-Jo and others. Other important investigations performed include RA factor, anti-cyclic citrullinated peptide (anti-CCP), creatine phosphokinase (CPK), aldolase, angiotensin-converting enzyme (ACE), and electromyography (EMG). 

Pulmonary manifestations like nonspecific interstitial pneumonia (NSIP), pleural effusion, usual interstitial pneumonia (UIP), interstitial pneumonitis, hypersensitivity pneumonitis, pulmonary arterial hypertension (PAH), tuberculosis, and pulmonary embolism were seen. Pulmonary hypertension was assessed on echocardiography and tuberculosis was diagnosed on the basis of sputum acid-fast bacillus (AFB) and nutrigenomic amino-acid therapy (NAAT) test (GeneXpert Dx system, Cepheid, Sunnyvale, CA). In addition to this, treatment history and immunological profile of these patients were recorded. Statistical analysis was performed using IBM Statistical Package for the Social Sciences; version 23 (SPSS Inc., Chicago, IL). Mean ± standard deviation (SD) was calculated for age and duration of disease. While gender, pulmonary manifestations, CTDs, medication used, and immunological tests were described in terms of frequency and percentages. Inferential statistics were explored using independent t-test and chi-square test. P-value ≤0.05 was taken as significant.

## Results

A total of 290 patients with rheumatologic disorders were included in this study, out of which 90.7% (263) were females and mean age of the entire cohort was 35.13 ± 12.53 years. A majority, 71% (206), of the patients were young (≤ 40 years of age). RA was the most commonly observed rheumatologic disorder in this cohort which was in 70.3% (204) patients. RA was more prevalent in patients of above 40 years of age as compared to young patients (≤ 40 years of age) with a frequency of 81% vs. 66%; p=0.012. Similarly, the second most common rheumatologic disorder was SLE which was observed in 14.1% (41) of the total cohort. SLE was found to be more associated with young patients (≤ 40 years of age) as compared to patients above 40 years of age with a frequency of 18.4% vs. 3.6%; p<0.001. The demographic characteristics and distribution of rheumatologic disorders are presented in Table 1.

**Table 1:** Demographic characteristics and rheumatologic disorders *significant at 5% SD: standard deviation.

Characteristics	Total	Age		p-value
		≤ 40 years	> 40 years	
N	290	206	84	-
Age(years)				
Mean ± SD	35.13 ± 12.53	28.56 ± 7.09	51.25 ± 7.26	<0.001*
Maximum - Minimum	80 - 13	40 – 13	80 - 41	
Gender				
Male	9.3% (27)	11.2% (23)	4.8% (4)	0.021*
Female	90.7% (263)	88.8% (183)	95.2% (80)	
Rheumatologic disorders				
Systemic lupus erythematosus (SLE)	14.1% (41)	18.4% (38)	3.6% (3)	<0.001*
Rheumatoid arthritis (RA)	70.3% (204)	66% (136)	81% (68)	0.012*
Mixed connective tissue disease (MTCD)	4.8% (14)	6.3% (13)	1.2% (1)	0.065
Overlap Syndrome	0.7% (2)	0.5% (1)	1.2% (1)	0.510
Dermatomiyositis	0.7% (2)	0.5% (1)	1.2% (1)	0.510
Vasculitis	0.3% (1)	0% (0)	1.2% (1)	0.117
Scleroderma	9% (26)	8.3% (17)	10.7% (9)	0.506
Sjogren syndrome	0.3% (1)	0.5% (1)	0% (0)	0.522
Duration of disease (years)				
Mean ± SD	3.87 ± 4.3	3.19 ± 3.17	5.56 ± 5.95	<0.001*
Maximum - Minimum	30 - 0.1	20 - 0.1	30 - 0.2	

Clinical investigations and their respective results are presented in Table 2. Positive results in the investigation of RA factor were observed in 65.9% (191) of the patients, anti-CCP in 37.9% (110), ANA in 31.4% (91), anti-dsDNA in 18.6% (54), anti-Sm in 10% (29), anti-RNP in 6.6% (19), and anti-Ro in 6.2% (18) of the patients.

**Table 2:** Clinical investigations

Clinical investigations	Investigated	Positive Results
Antinuclear antibody (ANA)	100% (290)	31.4% (91)
Anti-double stranded DNA antibody (anti-dsDNA)	100% (290)	18.6% (54)
Rheumatoid (RA) factor	100% (290)	65.9% (191)
Anti–cyclic citrullinated peptide (anti-CCP)	100% (290)	37.9% (110)
Anti-Smith (anti-Sm)	100% (290)	10% (29)
Ribonucleoprotein antibodies (anti-RNP)	100% (290)	6.6% (19)
Anti-Ro	100% (290)	6.2% (18)
Anti-La	99.7% (289)	4.2% (12)
High serum titers of antitopoisomerase 1 (anti-Scl-70)	99.7% (289)	12.8% (37)
Anti-Mi-1	99.3% (288)	0% (0)
Antineutrophil cytoplasmic (cANCA)	99.3% (288)	0.3% (1)
Antineutrophil cytoplasmic (pANCA)	99% (287)	0.3% (1)
Anti-Jo	74.8% (217)	0.5% (1)
Creatine phosphokinase (CPK)	63.8% (185)	0% (0)
Aldolas	21.4% (62)	0% (0)
Angiotensin converting enzyme (ACE)	3.1% (9)	0% (0)
Electromyography (EMG)	0.3% (1)	0% (0)

The distribution of pulmonary manifestations among the selected cohort of patients is presented in Table 3. Pulmonary manifestations were observed in 27.2% of the patients while the reaming 72.8% (211) were free from pulmonary manifestations. PAH was the most commonly observed disease which was observed in 11.4% (33) of the patients followed by NSIP which was observed in 9.3% (27) of the patients. Among the lesser common, pleural effusion was 5.5% (16), UIP was 4.8% (14), interstitial pneumonitis was 3.8% (11), and tuberculosis was 1.7% (5), while hypersensitivity pneumonitis and pulmonary embolism were observed in one patient (0.3%) each. No statistically significant association was found between the age groups and distribution of patterns of pulmonary manifestations.

**Table 3:** Patterns of pulmonary manifestations

Patterns of pulmonary manifestations	Total	Age		p-value
		≤ 40 years	> 40 years	
N	290	206	84	-
Pleural Effusion	5.5% (16)	5.8% (12)	4.8% (4)	0.719
Interstitial Pneumonitis	3.8% (11)	4.4% (9)	2.4% (2)	0.421
Usual interstitial pneumonia (UIP)	4.8% (14)	4.9% (10)	4.8% (4)	0.973
Nonspecific interstitial pneumonia (NSIP)	9.3% (27)	8.3% (17)	11.9% (10)	0.332
Pulmonary arterial hypertension (PAH)	11.4% (33)	12.6% (26)	8.3% (7)	0.297
Tuberculosis (TB)	1.7% (5)	1.5% (3)	2.4% (2)	0.583
Hypersensitity Pneumonitis	0.3% (1)	0% (0)	1.2% (1)	0.117
Pulmonary Embolism	0.3% (1)	0.5% (1)	0% (0)	0.522
Normal	72.8% (211)	72.8% (150)	72.6% (61)	0.973

Medication therapies received by the patients are presented in Table 4. Almost all of the patients, 99.3%, were on steroids, 90% (261) were receiving hydroxychloroquine therapy, and 67.2% (195) were receiving methotrexate.

**Table 4:** Medication therapies received by the patients **Excluding 10 missing values i.e. 280 = 191 (≤ 40 years) + 81 (> 40 years) *significant at 5%

Treatment	Total	Age		p-value
		≤ 40 years	> 40 years	
N	290	206	84	-
Steroid	99.3% (288)	100% (206)	97.6% (82)	0.026*
Methotraxate	67.2% (195)	66.5% (137)	69% (58)	0.676
Leflunamide	2.8% (8)	1.5% (3)	6% (5)	0.034*
Salazopyrin	34.5% (100)	30.6% (63)	44% (37)	0.029*
Hydroxychloroquine	90% (261)	91.3% (188)	86.9% (73)	0.262
Biologics	0.3% (1)	0% (0)	1.2% (1)	0.117
Mycophenolate Mofetil (MMF)	6.2% (18)	8.3% (17)	1.2% (1)	0.024*
Azathioprine (AZA)	16.6% (48)	19.4% (40)	9.5% (8)	0.04*
Cyclosporine**	1.4% (4)	1% (2)	2.5% (2)	0.373
Nonsteroidal anti-inflammatory drugs**	79.6% (223)	76.9% (153)	86.4% (70)	0.215
Cyclophosphamide**	0.4% (1)	0.5% (1)	0% (0)	0.514
Aspirin**	15.7% (44)	15.6% (31)	16% (13)	0.970
Calcium channel blockers (CCB)**	16.1% (45)	16.6% (33)	14.8% (12)	0.617
Sildenafil**	10.4% (29)	12.1% (24)	6.2% (5)	0.118
Bosentan**	1.8% (5)	2.5% (5)	0% (0)	0.142

The distribution of rheumatologic disorders by the presence and absence of pulmonary manifestations are presented in Table 5. The presence of pulmonary manifestation was found to be associated with SLE, mixed connective tissue disease (MTCD), scleroderma, overlap syndrome, and dermatomyositis, while, it was found to be lesser associated with RA.

**Table 5:** Distribution of rheumatologic disorders by the presence and absence of pulmonary manifestations *significant at 5%

Rheumatologic disorder	Total	Pulmonary manifestations		p-value
		Absent	Present	
N	290	211	79	-
Systemic lupus erythematosus (SLE)	14.1% (41)	9% (19)	27.8% (22)	<0.001*
Rheumatoid arthritis (RA)	70.3% (204)	86.3% (182)	27.8% (22)	<0.001*
Mixed connective tissue disease (MTCD)	4.8% (14)	0.9% (2)	15.2% (12)	<0.001*
Overlap Syndrome	0.7% (2)	0% (0)	2.5% (2)	0.02*
Dermatomyositis	0.7% (2)	0% (0)	2.5% (2)	0.02*
Vasculitis	0.3% (1)	0% (0)	1.3% (1)	0.102
Scleroderma	9% (26)	3.3% (7)	24.1% (19)	<0.001*
Sjogren syndrome	0.3% (1)	0.5% (1)	0% (0)	0.540

## Discussion

In this study, we have presented the prevalence and distribution of pulmonary manifestations in patients with CTD presenting at a tertiary care hospital of Karachi, Pakistan. In our cohort of patients with CTD, RA was the most common rheumatologic disorder observed in 70.3% followed by SLE which was observed in 14.1% of the patients. In this cohort, pulmonary involvement was observed in 27.2% of the patients with a leading share of PAH (11.4%) and NSIP (9.3%).

Pulmonary complications are reported as the initial clinical manifestation for a significant number of CTD cases [14-16]. Its prevalence has been reported in around 12% to 34% of the CTD cases [14,16-20]. The observed prevalence of 27.2% in our study is aligned with findings of these past studies, however, pulmonary involvement has been reported in as high as 67.1% in the subset of CTD patients [21]. It can be observed in almost all types of CTD such as RA, SLE, scleroderma, dermatomyositis, Sjogren syndrome, etc. [22]. Involvement of the lung, mostly in the form of ILD, in patients of CTD has an adverse impact on outcomes with poor prognosis and a substantial increase of risk of mortality [7,21]. ILD was reported to be associated with about 10% increase in mortality rate in patients with RA [23,24].

However, pulmonary hypertension of primary nature is not so common in patients with RA. In the study carried out currently, the percentage of those patients who were found to have pulmonary hypertension was only 11% [25].

A study conducted by Badui et al. documented an occurrence rate of 9% of pleural effusions in patients suffering from SLE [26]. In a different study that utilized the results from HRCT, the rate of occurrence was found to be around 10%. In the study being carried out currently, pleural effusions were documented to be present in around 15% of the patients who were suffering from SLE.

Both clinical manifestations, as well as histology of ILD, may vary over a wide range; clinical manifestations can be merely complaint of marginal symptoms or as severe as respiratory failure, similarly, histology can vary from simple inflammation to higher grade fibrosis [27]. Due to the wide ranges of clinical presentation, often with no obvious signs and symptoms and negative pathophysiological examinations, the clinical accuracy is sub-optimal for the diagnosis of ILD associated with CTD [21]. The most common types of pulmonary manifestations in patients with CTD are PAH, NSIP, UIP, desquamative interstitial pneumonia (DIP), organizing pneumonia (OP), and lymphocytic interstitial pneumonia (LIP) [27]. PAH and NSIP were the most commonly observed pulmonary manifestations in our study; the presence of pulmonary manifestation was found to be associated with SLE, MTCD, scleroderma, overlap syndrome, and dermatomyositis, while, it was found to be lesser associated with RA.

Although the pathological and clinical presentation of pulmonary manifestations associated with CTD is the same as that of other rheumatologic disorders, their management, treatment, and prognosis differ to a great extent [22]. Therefore, an extensive clinical workup is needed for the diagnosis of ILD associated with CTD, which includes history, physical examination, invasive and non-invasive, serologic testing and lung biopsy (when needed) [7,22]. Due to the inherent complexities, heterogeneity in clinical manifestation, and scarcity of data and well-designed protocols guidelines, the management of CTD patients with ILD is a clinical dilemma [7,28]. Therapeutic options available for the management are very limited and mostly very toxic [28]. Hence, collaborative efforts are needed in designing a multidisciplinary pathological, radiological, and clinical diagnosis and management approach [7,22,27-30].

To sum it up, pulmonary manifestations are frequently found in several CTDs at some juncture during the tenure of the disease. Unfortunately, a few of these manifestations may go undetected as they may not show any symptoms such as interstitial fibrosis which may gradually progress in the patient eventually resulting in a fatality. Thereby, it is essential to recognize the disease in its early stages and introduce therapy and treatment accordingly which may aid in lowering the rates of death and morbidity amongst patients.

Although this study was conducted at one of the largest public-sector hospitals of Karachi, single-center experience and observational nature of the study are the key limitation of this study.

## Conclusions

Pulmonary manifestations in patients with CTDs are not that uncommon (27.2%). PAH and NSIP collectively account for three-fourth of the total prevalent cases of pulmonary involvement in CTD patients. Further studies are needed to elucidate the incidence, prevalence, diagnosis, and management of this often life-threatening condition.
